# Transactions of the Provincial Medical and Surgical Association

**Published:** 1836-04

**Authors:** 


Art. XIII.—*Transactions of the Provincial Medical and Surgical Association.*—London and Worcester. Vol. I. II. and III., 1833, 1834, and 1835. 8vo. *Plates.*

These volumes have been already noticed in several Journals and Reviews; but, as they are the production of an Association recently formed, the proceedings of which are, each successive year, assuming more importance, whether regarded in respect of what has been actually accomplished, or of the promise which it affords of increasing utility, we are induced to give a brief retrospect of the progress of the institution, and a condensed analysis of the Transactions.

The Provincial Medical and Surgical Association was instituted at Worcester, in the year 1832, under the auspices of the venerable Dr. Edward Johnstone, of Birmingham, its first president. Upon this occasion the fundamental rules and regulations of the society were agreed upon, the plan upon which it should be conducted marked out, and the Association completely organized. It has been stated that the Provincial Association took its rise from, and was constituted upon, the model of the British Association for the Advancement of Science. This statement, however, we have reason to believe, is unfounded. The truth appears to be, that the Provincial Medical Association owes its origin, as we are informed by its founder, Dr. Hastings,* to the manner in which the Midland Medical and Surgical Reporter, published at Worcester during the years 1828-32, was supported by the members of the profession resident in the midland counties. The editors of that journal, and especially Dr. Hastings, conceived the idea that it would be practicable to form an institution in the provinces, similar in its objects to the Medico-Chirurgical Societies of London and Edinburgh; and it is to the active, zealous, and unwearied exertions of Dr. Hastings in carrying this

* The Address delivered at the Anniversary Meeting, &c., p. 94. See Note.

idea into operation, that the Provincial Association owes not only its original formation, but also the present high station which it occupies among the established institutions of the country. If we are to go deeper into the question of the origin of this and similar societies, which have lately sprung up in this country and elsewhere, we must look to the recent progress of the human mind in the acquirement of knowledge, and to the spirit of the age in which we live. It is the combined operation of these causes which alone affords the true explanation of the similarity of plan found to exist in institutions, the objects of which are in many respects similar; and it is surely not surprising that, where the intellectual constitution of man is in itself the same, acts under the same circumstances, and is stimulated to exertion by similar wants, there should be a unity of plan as well as of purpose in the proceedings instituted for the supply of these wants. The actual state of science, whether general or medical, and the effect produced upon the minds of those engaged in scientific pursuits by the peculiar constitution of the times, alike demand and favour the co-operative system, and, in the one instance as well as in the other, produced like results,—the formation of associations fitted for the purposes contemplated. These were nearly simultaneous in point of time, but, we believe, perfectly distinct, and at first uninfluenced by each other.

The organization of the Provincial Association seems well calculated to ensure that measure of success which its acknowledged utility, and the importance of the objects contemplated by it, so justly merit. The management of the affairs of the institution is vested in a president, vice-presidents, secretaries, and council. That the prosperity of such societies as this is mainly dependent upon the manner in which the trust reposed in its managers is fulfilled is evident; and it will be found, upon examination, that much of the success which similar institutions have obtained has arisen from the activity and energy manifested by those who have the immediate care of their concerns. In this respect the Provincial Association has possessed singular advantages, in having for one of its most influential officers the same ardent cultivator of medical science to whom it owes alike its origin and advancement. It is not too much to say, that the astonishing progress made by the Association in the short period of time which has elapsed since its formation is mainly attributable to the energy and zeal of its secretary, Dr. Hastings; and it is no small tribute of praise to the council in general, as well as to those especially concerned in drawing up the constitution of the society, that the original plan works so well under their superintendence as to have required no alteration in the fundamental laws and regulations.

The most important objects originally contemplated by the Association are, the assembling together of the members at the anniversary meetings,—the delivery of an address at their meetings,—and the collecting and publishing of valuable information under the form of Transactions. Numerous advantages result from the assembling together of the members of the profession at the anniversaries. In addition to the first general meeting at Worcester, in which the society was constituted, three such meetings have been now held: one in 1833, at Bristol, under the presidency of Dr. Carrick; one in 1834, at Birmingham, under Dr. John Johnstone; and one in 1835, at Oxford, at which the Regius

Professor of Medicine in that university presided. At each of these meetings the gratification experienced from the cultivation of mutual kindly feelings in the members of the profession, from the intermingling with so many of the estimable and eminent men that adorn at the same time that they receive honour from the noble science of which they are the cultivators, and from meeting with those amongst whom former habits of intimacy had existed, and the kindling anew those sympathies of friendship the remembrance of which had been cherished throughout years of absence, but which, but for these meetings, might never again have been called forth, must have come home to most of those who have attended them. At the same time, the tone given by the superior minds thus congregated together for the promotion of objects which all have in view, is communicated in a measure to each member of the Association; for who is there, while engaged in the contemplation of what has been effected by the patient research, industry, and talent of others, and seeing the consideration in which those who have obtained for themselves a lasting memorial of honour in the annals of our science are held, but feels himself excited to renewed exertions in his appointed course, and seeks with fresh ardour to avail himself of the advantages, natural or acquired, of which he may be possessed, or which he can command.

The addresses delivered at the general meetings are well fitted to inculcate and foster these principles. That of Dr. Hastings, read at the opening of the Association, drew the attention of the members to many important points of enquiry upon which the present state of our knowledge is extremely defective; a careful examination and comparison of the facts which we already possess, and the observation of new ones, being requisite for their further elucidation. The addresses of Drs. Barlow and Conolly, delivered at Bristol and Birmingham, and published in the second and third volumes of the Transactions, with that of Dr. Prichard, recently delivered at Oxford, by pointing out the actual progress made during the preeeding years, contribute much to the attainment of the same important ends. But these addresses are not only valuable in thus directing our attention to the existing state of medical science, and to the means of remedying such deficiences as may be found; they are also valuable historical records, and, considered in relation to what may be termed the literature of medicine, (if drawn up in future upon the same plan,) will form a series of no ordinary interest.

The papers published in the volumes of the Transactions of the Association are necessarily of various degrees of merit; but, upon a careful examination, we think all of them creditable to the industry and zeal of their several authors, while some are of a very superior description, and in every respect worthy of the Association. The several communications published by the society may be arranged under the heads of, 1st, Addresses and Reports delivered at the Anniversaries; 2d, Reports of Infirmaries, &c.; 3d, Medical Topography; 4th, Medical Jurisprudence; 5th, Miscellaneous Essays and Cases; and 6th, Biographical Memoirs. These divisions are adopted in the volumes of Transactions, although not in the same order of arrangement. Under the first head we have, in addition to the Addresses to which reference has already been made, two Reports read at the Birmingham meeting,—one on the Chemistry of the Blood, as illustrative of Pathology, by the late lamented Mr. Jennings, of Leamington; and the other on the Present State of our Knowledge of Anatomy, by Mr. Turner, of Manchester. It is not our intention to give more than a passing notice of these papers, but we should not do justice to the authors did we omit to direct the attention of our readers to the important observations upon the varied states of the blood in different diseases, more especially in fevers, inflammations, and chlorosis; and to the tables by which they are accompanied in Mr. Jennings' report; and to Mr. Turner's classification of the elementary tissues of the human body.

The Reports of Infirmaries and Dispensaries, we regret to observe, with the exception of one on the State of Disease in the City of Worcester during the year 1832, by Dr. Streeten, and a valuable statistical document from Van Dieman's Land by Mr. Scott, are confined to Birmingham. This is a department of the Transactions of the Association which might be extended with great advantage, and perhaps there is no information which could be communicated more acceptable to the profession, and none more fitting for the Transactions of a Provincial Medical Society, than a series of reports of this description from the physicians and surgeons of the Medical Institutions throughout the country. It is in this and the succeeding department of enquiry, namely, Medical Topography, that the Association can render most valuable assistance, by filling up deficiences in a branch of the science of medicine hitherto but little investigated; and the profession in general are entitled to expect, at the hands of provincial physicians, that they will not allow this important portion of the wide field of medical science to remain unbroken and uncultivated. We trust, therefore, that the future volumes of the Transactions will be enriched with many such communications as those that have appeared in those already published.

That the department of Medical Jurisprudence is also a branch of knowledge which may be advantageously cultivated by the provincial profession, we have evidence afforded us in two interesting communications in the first and third volumes of the Transactions. The first of these is a case of suspected Poisoning by Cantharides, reported by Dr. Hastings; the other the celebrated Bristol case of poisoning by sulphuret of arsenic, in which disinterment of the body took place; related and commented upon by Dr. Symonds.

The next division, that of Miscellaneous Essays and Cases, occupies the greater portion of these volumes, and combines many very valuable papers. Among the most important of these are, in the first volume, Dr. Barlow's Essay on the Objects and Modes of Medical Investigation, and the Observations on Strangulated Hernia, by Mr. James; in the second, the Observations on the Effects of Strychnine in some forms of Paralysis, by Dr. Bardsley; and those upon Epidemic Influences, &c., by Dr. Brown; and in the third, an Essay on Puerperal Convulsions, by Mr. Ingleby. Of the individual Cases, Dr. Traill's Case of Hydrocephalus; the Case of Melanosis, by Dr. Williams; one of Aneurism of the Basilar Artery, by Mr. Jennings; and that of Osteo-Sarcoma, by Mr. Hetling, in the first volume: the Cases of Hydrophobia by Mr. Grindrod; of Uterine Hydatids, by Mr. Watson; and of Injury of the Heart, by Mr. Davis, in the second: and Mr. Selwyn's Case of Extra-Uterine Fœtation, in the third,—are among the most interesting. Several of these are accompanied with beautiful and highly finished lithographic engravings. We have only to regret that our limits will not allow us, upon the present occasion, to enter at length into the merits of these communications, and of the contents of the volumes in general.

The Biographical Memoirs of Drs. Thackaray, Darwall, and Jackson, are ably written, and contain many interesting particulars of the lives of these eminent individuals. The contemplation of the progress towards the attainment of the high honours of our profession by the worthies which adorn its annals is fraught with instruction; and there are few perhaps who can behold successive difficulties perseveringly encountered, and the most formidable impediments surmounted by the struggles of an ardent mind, without feeling stimulated to follow on in the same bright course, and endeavouring, each for himself, to work out according to the measure of his ability and the opportunities afforded him a title to that gratitude, to that imperishable record of honour, which a successful cultivation of the enlarged and beneficent science of medicine cannot fail to secure. The Provincial Association must necessarily contribute much to the development of such a spirit among its members, and is admirably calculated to inculcate liberal views of science, to promote co-operation in the observing and recording of facts, and to foster that noble species of emulation which, divested of every jealous or party feeling, seeks only to confer real and lasting benefit upon suffering humanity. That the Association is in accordance with the spirit of the times is abundantly evidenced by the success which has attended it. In the short space of four years, it numbers 567 individuals of the medical profession among its members, including a large proportion of the most eminent physicians and surgeons residing in the provinces: it has been received with honour of no ordinary kind in the most ancient seat of learning in our land; the diploma of the university having been conferred upon two of its members on the occasion; and it has only now to proceed in the same course of usefulness, so auspiciously marked out in the commencement of its labours, to ensure that measure of prosperity which the high importance of its objects so justly entitle it to attain.

The recent meeting at Bury St. Edmund's of members of the profession in the Eastern counties, desirous of constituting themselves a branch of the Association, and offering themselves as fellow-labourers in the same field, is to be regarded as affording additional proof, were such wanting, of the activity, energy, and zeal, for the promotion of their science, which characterizes the members of the medical profession; at the same time that it is a valuable testimonial of the consideration in which the Association is generally held in this country.

